# Exploring the Antecedents and Consequences of Perceived Fairness in Beef Pricing: The Moderating Role of Freshness Under Conditions of Information Overload

**DOI:** 10.3390/foods14111844

**Published:** 2025-05-22

**Authors:** Kyung-A Sun, Joonho Moon

**Affiliations:** 1 Department of Tourism Management, Gachon University, Sungnam 13120, Republic of Korea; kasun@gachon.ac.kr; 2 Department of Tourism Administration, Kangwon National University, Chuncheon 24341, Republic of Korea

**Keywords:** beef, organic, price perception revisit intention, freshness, information overload

## Abstract

Organic labeling is a potentially influential factor in shaping consumer behavior toward beef products. However, limited research has examined consumer responses about perceptions of organic beef. This research thus explores the relationship between organic perception of beef, price fairness, and revisit intention. This research also investigates the moderating role of freshness in the impact of organic perception of beef on price fairness using information overload as a theoretical underpinning. An online survey targeted American consumers, with 415 responses collected via Clickworker. All participants were based in the United States regarding the consumption amount in the market. The Hayes Process Macro Model 7 was employed to test the research hypotheses. This research performed a median split analysis to scrutinize the moderating effect of freshness on the relationship between organic perception and price fairness. The findings indicate that the perception of organically produced beef positively affects price fairness and revisit intention. Furthermore, price fairness was found to influence revisit intention. The study also revealed a significant moderating effect of freshness on the relationship between organic perception of beef and price fairness. These outcomes contribute to the literature by clarifying the interrelationships among these four attributes within the context of beef products.

## 1. Introduction

Verified Market Research [1] reported that the organic beef market is projected to reach 19.7 billion US dollars, driven by increasing consumer concerns about health and the environment. This suggests that the organic beef market will continue to experience significant growth. Wang et al. [2] further emphasized that consumers are willing to pay a price premium for organic beef due to its perceived health benefits. Despite this market expansion, there remains a limited body of research exploring consumer behavior regarding organic beef. Previous studies have defined organic perception as the absence of chemical fertilizers and pesticides, noting that consumers tend to assign higher market value to organic products because they are perceived as safer focusing more on sustainable production processes [3,4]. Scholars argued that the importance of organic products has grown within the food industry, as consumers increasingly prioritize organic attributes [5,6]. However, there is a lack of focused research on the impact of organic perception specifically in relation to consumer behavior towards beef products. Given the growing importance of organic products, it is essential for businesses to develop adaptive marketing strategies to increase sales in the organic beef market. This study aims to fill this gap by examining consumer perceptions of the organic beef product category. Thus, the concept of organic perception is treated as the independent variable in this research. Putman et al. [7] also highlighted that beef is a primary food source in the United States, with an annual per capita consumption of 25 kg. This underscores the importance of focusing on US consumers as a key demographic for investigating consumer behavior.

This work selects revisit intention as the dependent variable because it is linked to vendors’ sales [8,9]. Moreover, the revisit intention might be more suitable for organic beef because places for purchasing beef could be more diversely chosen by consumers rather than repetitive purchasing. It suggests that visiting certain places to purchase beef is likely to become more important from the sellers’ perspective than repeated buying. Furthermore, this work chooses price fairness as a mediating variable because it can function as both an explanatory and an explained variable [10,11]. Wang et al. [2] noted that premium price is used as a marketing tool in the area of organic beef business, although the price level of beef for premium is hard to understand from the perspective of consumers. Also, the price of beef is not lower than that of other food ingredients [12,13]. It can be inferred that the price evaluation based on the utility and cost of beef might be an essential attribute in consumer decision-making.

The next focus of this study is the moderating effect of beef freshness. The extant literature addressed that freshness is an imperative element in food decision-making because it is linked to food safety and promoting individual health conditions [14,15]. Also, prior research noted that the freshness of food could be assessed by visual cues such as color and shape [16,17,18]. However, previous studies contend that offering excessive information may cause unwanted outcomes for businesses because individuals dislike complexity in their decision-making process [19,20,21]. The presentation of excessive product information can lead to adverse selection and suboptimal consumer outcomes, aligning with the theoretical framework of information overload and its detrimental effects on decision-making processes [20,22]. This study finds that an overabundance of product-related cues may impair consumer perceptions by increasing cognitive load. In particular, the notion of “organic” is often interpreted inconsistently by consumers, as it encompasses a broad array of attributes, including environmental sustainability, ethical production practices, chemical-free processing, and overall health benefits [5,23,24]. Owing to this conceptual ambiguity, the inclusion of additional product information, such as freshness claims, may not necessarily enhance clarity but instead further complicate consumer evaluations. From this perspective, supplementary information can become a cognitive burden rather than a decision aid. Accordingly, the present study investigates whether incorporating freshness cues into organic product descriptions enhances consumer responses, with a specific focus on perceived price fairness.

All things considered, the purpose of this research is to explore the relationship between organic and price fairness and revisit the intention of the decision-making of US consumers in purchasing beef. Another objective is to examine the moderating effect of freshness perception on the relationship between organic and price fairness in beef. This study investigates US beef consumers’ perceptions and behaviors using four key attributes: organic perception, price fairness, revisit intention, and freshness. Data were collected through an online survey, yielding a final sample of 415 respondents for statistical analysis. The central objective of this research is to assess the explanatory power of the information overload framework by testing the moderating effect of freshness on the relationship between organic perception and perceived price fairness. This study contributes to the literature by elucidating the interrelationships among these four variables. Despite beef being a staple in the American diet, prior research has largely neglected the role of information provision in shaping consumer behavior. By addressing this gap, the study underscores the significance of information overload in influencing consumer judgments and decision-making within the context of beef consumption. The findings provide valuable managerial implications, guiding beef industry professionals to enhance their marketing strategies and better align with consumer decision-making processes.

## 2. Review of Literature and Hypotheses Development

### 2.1. Organic Perception

Organic perception refers to consumers’ awareness of the absence of chemicals in the food production process [6,23]. Since beef production often relies on chemical-based feeding practices, consumers tend to place greater value on organic products [3,24]. According to Ahmed et al. [24] and Song et al. [5], organic perception is strongly linked to eco-friendly production methods, as these processes emphasize ethical practices, sustainability, and minimizing environmental impact. Researchers have suggested that consumers’ increasing concerns about their health influence the higher valuation of organic food [3,25]. Davis et al. [4] documented that organic beef is considered more nutritious and safer to consume, which likely leads to more positive consumer evaluations. Their findings indicate that organic beef provides a greater intake of beneficial fatty acids that promote human health. Furthermore, Revilla et al. [26] argued that organic beef is more appealing in the market than conventional beef products due to its superior nutritional value and environmentally sustainable production process. In a study of café customers, Song et al. [5] found that consumers’ perceptions of organic attributes significantly influenced their evaluations of coffee, suggesting that similar effects may extend to organic beef in retail settings. Supporting this notion, Napolitano et al. [27] demonstrated that organic perception plays a pivotal role in beef purchase decision-making. A review of the literature indicates that the concept of “organic” is multifaceted, often encompassing attributes such as environmental friendliness, health benefits, product safety, and sustainability. These dimensions provide a foundation for examining how consumers perceive organic claims, specifically in the context of beef products.

### 2.2. Price Fairness

Price fairness is defined as how consumers rationally assess the prices of goods [28,29]. Consumers may perceive a price as unfavorable, and numerous studies have explored both the antecedents and consequences of price fairness [10,11]. For example, Stiawan et al. [30] found that price fairness significantly influences consumer loyalty to fashion products, as fair pricing reduces the economic burden from the consumer’s perspective. Putu and Ekawati [31] demonstrated that the perceived fairness of a price is influenced by product quality, with price acting as an indicator through which consumers evaluate their consumption experience. In a study of low-cost carrier customers, Atmaja and Yasa [10] identified price fairness as both an antecedent and a consequence of consumer decision-making, highlighting its potential role as a mediating variable. Similarly, Thies et al. [32], focusing on beef consumers, found that demand for beef is significantly influenced by consumers’ perceptions of price. These findings suggest that price fairness may serve as a critical mediator in shaping consumer behavior. Drawing from the prior literature, it is reasonable to infer that price fairness likely functions as a mediating variable within the context of organic beef consumption, particularly concerning how consumers process product information and form purchase intentions.

### 2.3. Revisit Intention

Revisit intention refers to the likelihood of an individual returning to a particular location for shopping, which is often considered a measure of customer loyalty [33,34,35]. Numerous scholars have explored revisit intention, as repeated visits are known to boost vendor sales [8,9,36]. For instance, Rajput and Gahfoor [37] used revisit intention as a key variable to examine consumer behavior in fast-food restaurants, highlighting that repeat visits contribute significantly to business revenue. Similarly, Al-Sulaiti [38] investigated the factors influencing revisit intention in the shopping mall context, arguing that retaining loyal customers is more cost-effective than acquiring new ones. Peng et al. [34] also studied revisit intention in cultural tourism, noting that repeat visits are linked to higher economic value, which supports tourism development. As a result, revisit intention has frequently been employed as a dependent variable in various research domains.

### 2.4. Hypotheses Development

Previous studies found that price is an important aspect in the evaluation of organic products [39,40,41]. Singh and Alok [41] contended that organic food evaluation is associated with an adequate price level. Konuk [36] uncovered the positive association between organic perception and price fairness by exploring consumers visiting organic food restaurants. This indicates that price fairness is likely to encourage organic food sales. De Toni et al. [42] alluded to price fairness as a critical attribute in the organic food business sector. In addition, the extant literature contends that organic perception positively influences revisit intention [36,43]. Konuk [28] also demonstrated the positive influence of organic perception on both price fairness and loyalty to a place for food consumption. Singh and Alok [41] also argued that organic perception leads consumers to visit stores again because of their value for better health conditions. Agnihotri et al. [44] and Chaturvedi et al. [45] observed that consumers are more likely to frequent establishments that offer organic products, particularly in the restaurant industry. Given that organic products symbolize values such as health, environmental consciousness, and sustainability, it can be assumed that consumers who align with these values are more willing to perceive higher prices as justified and fair. Additionally, rising living standards and growing interest in personal health suggest that increased awareness of organic attributes may act as a motivating factor for repeat visits. Based on these insights, this study proposes the following research hypotheses:

**Hypothesis** **1:** *Organic perception positively affects price fairness.*

**Hypothesis** **2:** *Organic perception positively affects revisit intention.*

Scholars disclosed the positive effect of price fairness on revisit intention [35]. Lai et al. [46] revealed that price perception plays a significant role in building the repurchase intention for medical services. Hride et al. [47] disclosed a positive relationship between price fairness and loyalty in the domain of online shopping. Similarly, Hasan [48] demonstrated a positive association between repurchase intention and price fairness in the case of Chinese restaurants. Halimi et al. [35] explored Halal food customers, and the results indicated that price fairness exerts a positive effect on revisit intention. Based on the literature review, this research proposes the following hypothesis:

**Hypothesis** **3:** *Price fairness positively affects revisit intention.*

### 2.5. The Moderating Effect of Freshness

Freshness refers to the state of recent production [14,15]. Food decays over time, and spoilage organisms deter consumers from eating spoiled food, such an aspect could be a perceived risk for food consumption [49,50]. As a solution, freshness is an important attribute for consumers because consumers guess the food safety from the freshness [15,50]. Previous research has shown that food freshness could be a clue to consumers regarding how delicious and nutritious certain foods are [49,50]. Moreover, consumers value fresh food because it promotes individual health conditions by minimizing the risk of foodborne illness [51,52]. Zeng et al. [51] addressed that consumers evaluate beef quality by its visual elements: color, blood, etc. The extant literature consistently identified freshness as a key attribute of food quality, particularly in the context of beef [53,54]. Liu et al. [55] emphasized the critical role of freshness in consumers’ evaluation of beef quality, which in turn influences their purchase decisions. Therefore, it is reasonable to infer that consumer decision-making is significantly affected by perceptions of freshness. Therefore, consumer decision-making is likely to be influenced by the degree of freshness. However, the price of beef varies depending on the conditions. Previous work also documented that beef is not a cheap ingredient; consumers are likely to feel a financial burden for the purchase of beef as compared to other meats: pork and chicken [56,57]. If beef is fresh and organic, consumers are likely to be overwhelmed by the price due to its likelihood of being expensive. In other words, emphasizing both organic perception and freshness together might cause a financial burden from the viewpoint of consumers, given its heuristics. Indeed, scholars have alleged that information overload causes undesirable business outcomes because consumers are more anxious due to the burden of information processing and adequate decision-making [19,20,21]. Prior studies demonstrated the effect of information overload in the context of the consumer behavior domain. For instance, Peng et al. [58] found that information overload causes adverse selection in the area of the online market. Also, according to Cheng et al. [59], information overload brings about negative consumer behavior in the case of electronic vehicle purchases. Aljanabi and AL-Hadban [60] additionally disclosed that consumers’ decision-making became less rational under the information overload condition in the context of green marketing. Wang et al. [2] additionally alleged that consumers have no convincing criteria for the premium price of organic beef. It implied that offering excessive information is likely to lead consumers to become overwhelmed. Thus, it is anticipated that the freshness of organic beef will be perceived as a luxury food item, which might undermine the perception of price fairness. To investigate this rationale, this study proposes the following hypotheses:

**Hypothesis** **4:** *Freshness significantly moderates the relationship between organic perception and price fairness of beef.*

**Figure 1:**
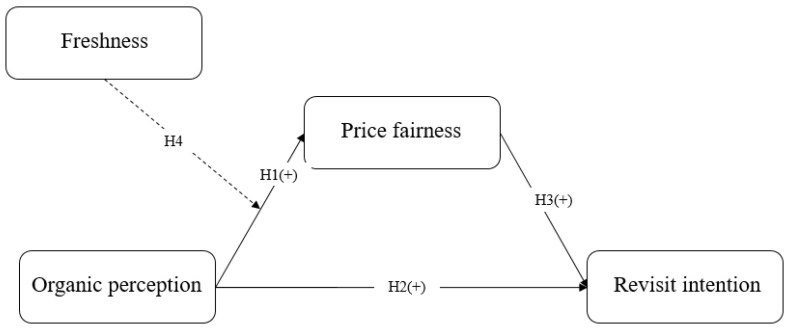
Research model.

By integrating the research hypotheses, this work presented the research model as follows:

Figure 1 illustrates the research model. Organic perception is the independent variable that exerts positive effects on both price fairness and revisit intention. Price fairness is a mediating variable. Price fairness is positively associated with revisit intention. Freshness significantly moderates the relationship between organic perception and price fairness.

## 3. Method

### 3.1. Description of Measurement Items

Table 1 presents information on the measurement items. A five-point Likert scale was used for measurement (1 = strongly disagree, 5 = strongly agree). This study referenced prior studies on the measurement of four attributes: organic perception [3,4,5], price fairness [11,30], revisit intention [34,38], and freshness [49,50,51]. The measurement items were adjusted to become more suitable for the objectives of the current research. Regarding the operational definition, organic perception is how beef is offered through an environmental process. Price fairness is defined as how consumers rationally assess beef costs. Revisit intention is defined as the likelihood of consumers visiting a place for beef shopping. Finally, freshness was defined as how consumers perceive beef to be pleasant to consume. All variables used four items other than revisiting intention. Revisit intention was measured by three items by referencing prior works [34,38].

### 3.2. Recruiting Survey Participants

The survey was the main instrument used. The survey was uploaded to Google’s platform. This work employed an online survey because the survey participants were less constrained by time and place. Data collection was performed using Clickworker (https://www.clickworker.com/, accessed on 1 October 2024). Clickworker has been commonly used in previous studies as an instrument for data collection [61,62,63]. Its popular use led this study to adopt the system as the main instrument for collection. Data were collected between 1 October and 7 October 2024, through a survey consisting of 415 American participants considering the beef consumption volume in the US market. Putman et al. [7] stated that beef is a main food source in the US; US consumers are likely to become the focal area for beef consumer research. The survey participants were compensated after the completion of the survey. It took less than five minutes not to lose focus on the survey responses. This research only collected the basic demographic information and perception of beef without personal information, such as identification number, which could become a category for the exemption of the Institutional Review Board (IRB). The number of observations is 415, and extant literature suggests that more than 250 observations are sufficient for statistical inference [64]. Table 2 presents the survey participant profiles. The number of males and females was 130 and 285, respectively. Approximately 68.7 percent of the survey participants were between 30 and 49 years old (30–39:139 (33.5%) and 146 (35.2%)). Table 2 also presents information on weekly beef consumption frequency (less than 1 time: 71, 1–2 time: 215, 3–6 times: 116, and every day: 13). Considering education, the number of students with less than a bachelor degree, bachelor’s degree, and graduate degree were 170, 172, and 73, respectively. Finally, 50.7 percent of the survey participants had a monthly household income below USD 5000.

### 3.3. Data Analysis

This work used frequency analysis to analyze the demographic information of the survey participants. This work adopts a 95 percent confidence interval level to appraise the significance of the parameters [65]. Exploratory factor analysis was then performed using varimax rotation. The loading cutoff value was 0.5. This study also applied a Cronbach’s alpha of 0.7 as the reliability test standard [65]. For testing the statistical significance of the model, this work used both the Kaiser–Meyer–Olkin Measure of Sampling Adequacy 0.7 and Bartlett’s Test of Sphericity χ^2^ [65]. This study also selected Eigenvalue 1 as the criterion for deriving the constructs [65]. Next, the mean and standard deviation (SD) of the variables were calculated. A correlation matrix was examined to scan the relationships between attributes. Moreover, this research adopted the ordinary least squares-based Hayes process model 7 using bootstrapping with 5000 to test the research hypotheses. Estimation using the Hayes process model is less likely to be biased because the normality assumption is not mandatory in the Hayes process analysis [66]. The moderating effect is examined by generating the attribute: Organic perception × Freshness. Then, this work ensures the moderating effect of freshness based on the slopes of the conditional effect of the focal predictor [66]. In addition, a median split analysis was conducted to examine the moderating effect of freshness. The median of the freshness was 4.75, and the median of the organic perception content was 3.00. Plus, this research performed the sensitivity analysis including gender and age as covariates, because such attributes might be able to affect the beef perception.

## 4. Empirical Results

### 4.1. Results of Validity for the Measurement Items

Table 3 presents the results of factor analysis. The Kaiser–Meyer–Olkin measure (0.857) and Bartlett’s Test of Sphericity Approx. Chi-square test (4946.887 (*p* < 0.01)); the results were statistically acceptable. All factor loadings were greater than 0.5, and Cronbach’s alpha values were greater than 0.7. Table 3 presents information on the following variables: organic perception (mean = 3.04; SD = 0.97), price fairness (mean = 2.83; SD = 0.99), revisit intention (mean = 4.33; SD = 0.88), and freshness (mean = 4.43; SD = 0.74).

### 4.2. Correlation Matrix and Results of Hypotheses Testing

Table 4 presents the results of the correlation matrix analysis. Revisit intention was positively correlated with price fairness (r = 0.219, *p* < 0.05), freshness (r = 0.543, *p* < 0.05), and organic perception (r = 0.309, *p* < 0.05). Price fairness was positively correlated with freshness (r = 0.115, *p* < 0.05) and organic perception (r = 0.375, *p* < 0.05). Freshness was positively correlated with organic content (r = 0.250, *p* < 0.05). The results indicated that revisit intention most strongly correlates with freshness.

Table 5 shows the results of the hypothesis testing. All models were statistically significant based on the F-values (*p* < 0.05). Organic perception had a positive effect on price fairness (β = 1.070, *p* < 0.05) and revisit intention (β = 0.106, *p* < 0.05). In sum, all hypotheses were supported. Price fairness had a positive impact on revisit intention (β = 0.237, *p* < 0.05). The results also showed a significant moderating effect of freshness on the impact of organic perception on price fairness (β = −0.156, *p* < 0.05). R^2^ values of model 1 and model 2 are 0.1598 and 1077. The values might indicate the relatively low explanatory power of the variable for price fairness and revisit intention. Gender appeared significant effect on revisit intention (β = 0.183, *p* < 0.05). The F-values for the test of interaction are also significant (*p* < 0.05).

**Figure 2:**
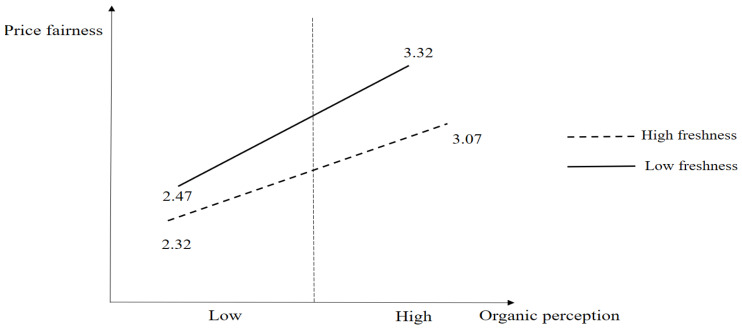
Results of the moderating effect of freshness. Note: Median _freshness_ = 4.75, Median _organic perception_ = 3.00.

Figure 2 presents the results of the median split analysis. The results show the mean values of the four groups (mean _low organic perception and low freshness_ = 2.47, mean _high organic perception and low freshness_ = 3.32, mean _low organic perception and high freshness_ = 2.32, and mean _high organic perception and high freshness_ = 3.07). The slope of the low freshness group (β = 0.445, *p* < 0.05) is steeper than the high freshness group (β = 0.288, *p* < 0.05) from the results of Table 5. It can be inferred that offering excessive information, such as freshness, is likely to deter consumers from building price fairness from the organic perception of beef. The F-value for the test of interaction is also significant (*p* < 0.05).

## 5. Discussion

Regarding the mean value of organic perception (mean = 3.04), consumers might perceive that organic beef is insufficient to consume because organic beef might be scarce and not cheap. In addition, the lowest mean value for price fairness indicates that beef prices are not sufficiently low. Additionally, the high mean value of revisit intention indicated that consumers might need to visit the store to purchase beef again because it is related to the health condition of daily life, as a sort of habitual consumption. It can be inferred that beef might be regarded as a necessary good based on the perception of survey participants. Consumers positively appraised beef’s freshness (mean = 4.43). Also, the correlation matrix suggested that organic perception is likely to become a more critical attribute for price fairness. Moreover, the correlation matrix revealed a positive association between organic perception and freshness, suggesting that consumers tend to perceive organic beef as fresher. However, the relatively low mean score for price fairness (mean = 2.83) indicates a degree of consumer skepticism regarding the fairness of beef prices. This may reflect a broader perception of beef as a relatively high-priced food product.

The results of the hypothesis testing also indicated that the organic perception of beef led consumers to elevate their perception of price fairness and revisit intention. It can be inferred that the organic perception framework of beef could become an appealing point for price fairness and revisit the intention of consumers. The findings aligned with Konuk [28] and Konuk [36] results, given the significance and positive influence of the organic perception of food. It can be inferred that organic is appealing to consumers in the context of the food business. In addition, the results revealed a positive relationship between price fairness and revisit intention. This implied that the consumers’ adequate price perception of beef led consumers to find a store again to purchase beef. Namely, offering affordable prices might become a key to accomplishing sustainable business through repeated visits of consumers. Also, the results externally validated the findings of Halimi et al. [35], implying that price fairness is critical for consumer decision-making because of the price sensitivity of food.

The results showed a significant moderating effect of freshness on the relationship between organic perception and price fairness. These results indicate that emphasizing the organic perception and freshness of beef together had an adverse impact on price perception. It is possible that consumers feel a price burden for beef in terms of both freshness and organic aspects. In other words, consumers might benefit sufficiently only from an organic perception of beef. Alternatively, the findings might indicate that the concept of “organic” is interpreted in diverse ways by consumers, encompassing multiple attributes such as health, safety, and environmental impact. As a result, the addition of freshness-related information could contribute to cognitive overload, complicating rather than facilitating consumer decision-making. This implies that presenting both organic and freshness cues simultaneously may be perceived as redundant or overwhelming. Moreover, the results indicated that female consumers are more likely to revisit the same locations for beef purchases. This pattern might be linked to the traditional role of women in meal planning and grocery shopping, which can foster stronger loyalty to specific retailers and result in more consistent shopping behaviors.

## 6. Conclusions

### 6.1. Theoretical and Practical Implications

The outcomes of this work contribute to the literature by revealing the relationship between four attributes: organic perception, price fairness, revisit intention, and freshness. Building on previous studies that emphasized the importance of organic perception in food appraisal [37,40,41,44], this study highlighted the significant influence of organic perception on both price fairness and revisit intention, based on an analysis of U.S. beef consumers using Hayes’ Process Macro Model 7. Moreover, this study confirmed the relationship between price fairness and revisit intention within the beef product sector [36,49]. These findings contribute to the literature by enhancing the external validity of consumer behavior theories in the context of food consumption. Furthermore, this work expanded the literature by revealing the moderating role of freshness in the relationship between organic perception and price fairness, providing deeper insight into the interactions among these three factors. Furthermore, this work demonstrated that excessive information can lead to adverse consumer outcomes [20,21], offering valuable implications for the marketing of beef products. By documenting the relevance of information overload theories in the beef consumption domain, this research sheds light on the literature, offering a broader understanding of consumer behavior in the case of beef products.

This study has some managerial implications. First, vendors of beef products might allocate more resources to attain organic products because organic beef could elevate both price fairness and revisit intention. This could be accomplished by allotting resources to managing suppliers. Through the thorough administration of suppliers, better organic beef products could be secured. Also, the sellers of beef might need to concentrate their effect on how to elaborate the organic aspect to the consumers because organic could be interpreted in varied ways from the viewpoint of consumers. Such communication might be accomplished by a certification mark from a reliable source. Namely, effective marketing communication is crucial for the sustainability of beef-related businesses. Sellers should prioritize emphasizing the organic aspects of their beef products in their messaging, particularly through packaging. However, an overemphasis on a wide range of product attributes could lead to consumer skepticism about the authenticity of organic claims, potentially resulting in a loss of market share in the retail sector. Additionally, an excessive amount of information may overwhelm consumers, leading to a heightened sense of price burden. In light of this, marketing managers should consider adopting a more conservative approach when presenting the benefits of beef products to avoid information overload. This approach would allow businesses to focus on the most relevant information to encourage consumer purchases. Moreover, beef sellers should allocate resources to maintain consistent supply conditions, as any perception of unfair pricing could harm customer loyalty. The findings also suggest that marketing strategies should target female consumers more specifically, as they exhibit stronger intentions to revisit specific retailers. Therefore, directing marketing efforts toward female consumers may offer a more efficient use of resources for beef sellers, potentially enhancing consumer retention and repeat purchases.

### 6.2. Research Limitations and Suggestions for Future Research

This research has some limitations. First, only four attributes were tested to examine beef consumers. This might become the reason for the relatively low R^2^ value. Future research might be able to contemplate more diverse attributes to investigate consumer behavior toward beef products. Moreover, the sample used in this work is limited to the US. Because beef prices and quality could vary depending on geographical conditions, future research might be able to consider different geographical cases to recruit survey participants. Regarding the profile of the sample, females were more. Also, most of the participants were relatively young (20s and 30s) because the data collection was implemented by an online survey. Future research might be able to collect data focusing more on both the male and older population because demographic attributes are likely to affect consumer behavior for beef. Such effort might be valuable in achieving an in-depth understanding of beef consumers.

## Figures and Tables

**Table 1:** Depiction of measurement.

Construct	Code	Item	Scale
Organic perception	OG1 OG2 OG3 OG4	The beef was produced in an environmentally. The beef was produced in organic manner. The beef was based on grass-fed. The beef production was eco-friendly.	Likert five-point scale 1: strongly disagree 5: strongly agree
Price fairness	PF1 PF2 PF3 PF4	The price of beef was fair. The price of beef was reasonable. The price of beef was acceptable. The price of beef was affordable.	Likert five-point scale 1: strongly disagree 5: strongly agree
Revisit intention	RI1 RI2 RI3	I intend to visit the place where I bought the beef again. I am going to visit the place where I purchased the beef again. I will revisit the store where I purchased the beef.	Likert five-point scale 1: strongly disagree 5: strongly agree
Freshness	FR1 FR2 FR3 FR4	The color of beef was important. The freshness of beef was essential. For me, the freshness of beef was critical. Fresh visuals of beef were imperative.	Likert five-point scale 1: strongly disagree 5: strongly agree

**Table 2:** Demographic information (N = 415).

Item	Frequency	Percentage
Male	130	31.3
Female	285	68.7
		
20–29 years’ old	60	14.5
30–39 years’ old	139	33.5
40–49 years’ old	146	35.2
50–59 years’ old	55	13.3
Older than 60 years’ old	15	3.6
		
Weekly eating frequency		
Less than 1 time	71	17.1
1–2 times	215	51.8
3–6 times	116	28.0
Everyday	13	3.1
		
Terminal academic degree		
Less than bachelor’s degree	170	41.0
Bachelor degree	172	41.4
Graduate degree	73	17.6
		
Monthly household income		
Less than USD 2500	103	24.8
Between USD 2500 and USD 4999	145	34.9
Between USD 5000 and USD 7499	78	18.8
Between USD 7500 and USD 9999	24	5.8
More than USD 10,000	65	15.7

**Table 3:** Results of factor analysis.

Construct	Code	Loading	Mean (SD)	Cronbach’s α	Eigenvalue	Explained Variance
Organic perception	OG1 OG2 OG3 OG4	0.785 0.842 0.795 0.862	3.04 (0.97)	0.870	3.135	20.903
Price fairness	PF1 PF2 PF3 PF4	0.884 0.945 0.907 0.864	2.83 (0.99)	0.938	5.624	37.496
Revisit intention	RI1 RI2 RI3	0.901 0.895 0.910	4.33 (0.88)	0.964	1.263	8.423
Freshness	FR1 FR2 FR3 FR4	0.703 0.807 0.871 0.793	4.43 (0.74)	0.840	1.868	12.455

Note: SD stands for standard deviation, the unit of explained variance is percent, total variance explained: 79.277, Kaiser–Meyer–Olkin Measure (KMO) of Sampling Adequacy: 0.857, Bartlett’s Test of Sphericity Approx. Chi-Square: 4946.887 (*p* < 0.01).

**Table 4:** Correlation matrix.

	1	2	3	4
1. Revisit intention	1			
2. Price fairness	0.219 *	1		
3. Freshness	0.543 *	0.115 *	1	
4. Organic perception	0.309 *	0.375 *	0.254 *	1

Note: * *p* < 0.05.

**Table 5:** Results of hypotheses testing: the moderating effect of freshness.

	Model 1 Price Fairness	Model 2 Price Fairness	Model 3 Revisit Intention	Model 4 Revisit Intention
	β (t value)	β (t value)	β (t value)	β (t value)
Constant	−0.033 (−0.05)	−0.022 (−0.03)	3.310 (21.56) *	3.067 (15.20) *
Organic perception	1.070 (4.57) *	1.066 (4.51) *	0.237 (5.25) *	0.247 (5.45) *
Freshness	0.393 (2.87) *	0.391 (2.85) *		
Interaction	−0.156 (−3.03) *	−0.155 (−2.98) *		
Price fairness			0.106 (2.39) *	0.106 (2.39) *
Gender		0.037 (0.38)		0.183 (2.06) *
Age		−0.012 (−0.26)		0.035 (0.86)
F-value	26.05 *	15.60 *	24.87 *	13.81 *
R^2^	0.1598	0.1602	0.1077	0.1188
Conditional effect of focal predictor				
Freshness				
4.00	0.445 (8.43) *	0.445 (8.34) *		
4.75	0.327 (6.52) *	0.329 (6.51) *		
5.00	0.288 (5.18) *	0.290 (5.18) *		
Index of mediated moderation	Index	Index		
	−0.0167 *	−0.0165 *		

Note: * *p* < 0.05, Interaction: Organic perception × Freshness in model 1 (Test of interaction: F = 9.18 *) Interaction: Organic perception × Freshness in model 2 (Test of interaction: F = 8.91 *).

## Data Availability

The original contributions presented in the study are included in the article, further inquiries can be directed to the corresponding author.
